# Shifting the balance of autophagy and proteasome activation reduces proteotoxic cell death: a novel therapeutic approach for restoring photoreceptor homeostasis

**DOI:** 10.1038/s41419-019-1780-1

**Published:** 2019-07-18

**Authors:** Yaoyan Qiu, Jingyu Yao, Lin Jia, Debra A. Thompson, David N. Zacks

**Affiliations:** 1 Department of Ophthalmology and Visual Sciences, University of Michigan Medical School, Kellogg Eye Center, Ann Arbor, MI USA; 2 Department of Ophthalmology, Xiangya School of medicine, The Second Xiangya Hospital, Central South University, Changsha, Hunan China; 3 Department of Biological Chemistry, University of Michigan Medical School, Ann Arbor, MI USA

**Keywords:** Autophagy, Protein folding

## Abstract

The P23H variant of rhodopsin results in misfolding of the protein, and is a common cause of the blinding disease autosomal dominant retinitis pigmentosa (adRP). We have recently demonstrated that degeneration of photoreceptor cells in retinas of P23H mice is due to the endoplasmic reticulum stress (ERS)-induced activation of autophagy that leads to a secondary proteasome insufficiency and activation of cell death pathways. We propose that this increased level of autophagy flux relative to proteasome activity, which we term the A:P ratio, represents a marker of altered photoreceptor cell homeostasis, and that therapies aimed at normalizing this ratio will result in increased photoreceptor cell survival. To test this postulate, we treated P23H mice with a chemical chaperone (4-phenylbutyric acid) to improve rhodopsin folding, or with a selective phosphodiesterase-4 inhibitor (rolipram) to increase proteasome activity. P23H mice treated with either of these agents exhibited reduced ERS, decreased autophagy flux, increased proteasome activity, and decreased activation of cell death pathways. In addition, rates of retinal degeneration were decreased, and photoreceptor morphology and visual function were preserved. These findings support the conclusion that normalizing the A:P ratio, either by reducing the ERS-induced activation of autophagy, or by increasing proteasome activity, improves photoreceptor survival, and suggest a potential new therapeutic strategy for the treatment of adRP caused by protein folding defects.

## Introduction

Inherited retinal degeneration (IRD) occurs in ~1 in 3000 people in the population and results from mutations in nearly 300 different genes^1,2^. This extreme genetic heterogeneity has complicated the development of therapies for individuals with IRD. Thus, there is an unmet need for therapies that target broadly shared pathophysiological mechanisms and that achieve lasting rescue in a mutation-independent manner. A significant subset of IRD-causing mutations result in misfolding, mistrafficking, or abnormal accumulation of proteins within photoreceptor cells, resulting in proteotoxic cell death, thus making the targeting of proteotoxicity an attractive therapeutic point of intervention for improving cell survival.

Rhodopsin, the visual pigment of rod photoreceptor cells, is synthesized and folded in the endoplasmic reticulum (ER) of the rod cell inner segment before its subsequent processing in the Golgi and trafficking to the outer segment^3^. Mutations in the rhodopsin gene *RHO*, a common cause of IRD, often lead to misfolding or mistrafficking of the rhodopsin protein, resulting in proteotoxicity^4^. A mutation that results in the substitution of histidine for proline at amino acid residue 23 of rhodopsin (RHO^P23H^; hereafter referred to as P23H) is the most frequent *RHO* mutation identified in the United States^1^. The P23H mutation has been extensively studied, and represents a prototypical model for studying proteotoxicity^5,6^.

In both the human disease and corresponding transgenic mouse models, misfolding of the rhodopsin P23H variant results in intracellular accumulation of mutant protein, increased endoplasmic reticulum stress (ERS), and aberrations in rod outer segment formation^7–11^. To deal with this increased ERS, cells can employ one of two clearance mechanisms, the ubiquitin–proteasome system or the autophagy–lysosome pathway^12–14^. Under normal homeostatic conditions, channeling the misfolded protein to either one of these two clearance pathways is protective. However, when protein stress is chronic, as in IRD, this intrinsic response is insufficient to prevent cell death.

Our previous studies examined the activity of autophagy in the P23H mouse retina and its role in photoreceptor cell degeneration^15^. We found that misfolded rhodopsin results in the persistent activation of autophagy that contributes to secondary proteasome insufficiency. We also showed that increasing autophagy in P23H mice further accelerates retinal degeneration, whereas inhibiting autophagy, either genetically or pharmacologically, improves proteasome levels and activity and reduces retinal degeneration. These findings highlight the reciprocal nature of autophagy and the proteasome, and suggest that the balance between their activity levels, which we term the A:P ratio, can serve as a marker of altered photoreceptor homeostasis.

In the present study we sought to further study the interdependency of ERS, autophagy and proteasome to determine whether shifting protein degradation from autophagy to proteasome can relieve proteotoxic ERS and improve cell survival. P23H mice were treated with either 4-phenylbutyric acid (4-PBA), a chemical chaperone that has been shown to reduce ERS by improving protein folding and shuttling to the proteasome degradation pathway^16–18^, or with rolipram, a selective phosphodiesterase-4 inhibitor that can act to directly increase proteasome activity levels^19–21^. Assays of ERS activation, proteasome activity and autophagy flux showed that both treatments resulted in normalization of the A:P ratio and increased photoreceptor cell survival and retinal function. These observations suggest that modulating the flux of misfolded protein from autophagy to the proteasome represents a potential therapeutic option for reducing proteotoxicity and improving cell survival.

## Methods

### Animals and experimental treatments

All experiments were performed following the Association for Research in Vision and Ophthalmology statement for ethical care and use of animals and the guidelines approved by the University Committee on use and Care of Animals at University of Michigan. The Rho^P23H/P23H^ mice were bought from Jackson lab, and crossed with C57BL/6J mice to produce Rho^P23H/+^ mice (P23H). The Rho^P23H/P23H^ mice were also crossed with green fluorescent protein (GFP)-LC3 (Riken laboratories, Tsukuba, Japan) mice to produce Rho^P23H/+^-GFP-LC3 mice. In all experiments on P23H mice, heterozygous mice were used, as this represents the most common clinical presentation. Mice were housed under standard conditions with 12 h of light and 12 h of dark in the vivarium of the University of Michigan Kellogg Eye Center.

4-phenylbutyric acid (PBA) (Santa Cruz, 200652) was dissolved in 0.9% saline at a concentration of 40 mg/ml and was given through daily intraperitoneal (IP) injections at a dose of 200 mg/kg from P14 up to 4 months. Control mice were given injections of vehicle alone (equivalent solution without 4-PBA).

Rolipram (Enzo Life Science, BML-PD175-0050) was dissolved in DMSO at a concentration of 250 mg/mL as a stock solution. A working solution at 0.5 mg/mL was diluted in 0.9% saline freshly prior injection. Mice were given rolipram at 5 mg/kg or equivalent vehicle alone intraperitoneally daily from P14 up to 4 months. Mice were monitored daily.

### Tissue collection

To control for the effect of autophagy flux, mice were given an IP injection of leupeptin (Sigma-Aldrich, L2884) (40 mg/kg body weight) 4 h before harvest^22,23^. All retinal samples were collected at the same time of the day, 1 p.m., to control for the time-dependent fluctuations in autophagy^24^. For better visualization of ubiquitinated proteins by western blot, mice were given an IP injection of MG132 (5 mg/kg) right after the last treatment of rolipram before collecting retinal samples.

Eyes were enucleated immediately after euthanizing mice. Retinas were harvested under a dissection microscope as previously described^24^. Briefly, after removing the cornea and lens to form the eyecup, the retina was dissected free of the retinal pigment epithelium.

### Chymotrypsin-like proteasome activity assay

An optimized assay was used for measuring chymotrypsin-like proteasome activity in the mouse retinas at 1 month of age using substrate Suc-LLVY-AMC (Enzo Life sciences, BML-P802-0005) as previously described^15^. Two retinas from each mouse were pooled as one sample. After adding 120 µl assay buffer (50 mM Hepes, pH 7.5, 20 mM KCl, 5 mM MgCl_2_, and 1 mM DTT), samples were sonicated and centrifuged at 4 °C at 10,000 g for 10 min to obtain cytosolic protein. Sixty micrograms of freshly prepared cytosolic protein and substrate Suc-LLVY-AMC were assessed in assay buffer containing 7 µM ATP (Dot Scientific, DSA30030-5) with or without the presence of the proteasome inhibitor lactacystin (Enzo Life sciences, BML-PI104-1000, Enzo Life Sciences) in a black-walled nine-well plate with a total volume of 250 µl in each well. An excitation wavelength of 380 nm and emission wavelength of 440 nm were used to scan the plate once per minute for 45 min in a Flexstation-II plate reader (Molecular Diagnostic). The readings at 40 min were used for analysis. All assays were repeated three times.

### Western-blot analysis

Retinas were sonicated in RIPA buffer (Thermo Scientific,89900) containing a protease inhibitor (Roche, 11697498001). After centrifugation, the supernates were collected and the protein concentrations were measured using a Bio-Rad RC DC Protein Assay Kit (Cat. No. 500-0119,0120,0121,0122). Equal amount of total protein was added to 4–15% SDS-PAGE (Tris-HCl Ready Gels; Bio-Rad Laboratories, 4561086) and transferred to polyvinylidene fluoride membranes (Bio-Rad Laboratories, 162-0177). After blocking with 5% milk in Tris-buffered saline, Bio-rad, 170-6435 contains 0.1% Tween 20 (Sigma, P7949) at room temperature for 1 h, the membranes were incubated with primary antibodies overnight at 4 °C. Primary antibodies used in this study include: LC3 (Cell Signaling technology, 4108S;1:1000), P62/AQATM1 (Novus biologicals, NBP1-48320S;1:1500), Beclin1 (Cell Signaling technology, 3495; 1:1000), p-SQSTM1 (Gene Tex, GTX128171; 1:400), Rho 4D2 (Novus biologicals, NBP1-48334; 1:2000), Ubiquitin (Cell Signaling, 3933; 1:1000), proteasome 20S (Enzo, BML-PW8155; 1:1000), GAPDH (Ambion Applied Biosystems, AM4300; 1:80000), RIPK3 (ABGENT, AP7819B, 1:1000), pRIPK3 (Abcam, ab195117, 1:1000), MLKL (ABGENT, AP14272, 1:2000), CHOP (Cell Signaling technology, 2895, 1:1000), BIP (Cell Signaling technology, 3177, 1:1000) and pMLKL (Abcam, ab196436, 1:1000). After washing, the membranes were incubated with secondary antibodies (Dako, P0447, P0448; 1:2000) at room temperature for 1 h. Blots were developed using SuperSignal West Dura Sunstrate (Thermo Scientific, 34075). Quantitative densitometry of the bands was measured by ImageJ. All experiments were repeated at least three times.

### Soluble and insoluble rhodopsin fractionation assay

Two retinas were pooled and lysed in 250 µl of ice-cold lysis buffer (PBS, pH 7.5, 5 mM EDTA, 1% TX-100) with protease inhibitor (Roche, 11697498001) for 30 min on ice with occasionally mixing. After centrifugation at 13,000 × *g* for 15 min, the supernate was collected as the soluble fraction. To prepare the insoluble fraction of the protein, the pellet was solubilized in 50 µl of 1% SDS in PBS for 10 min at room temperature. After additional 75 µl lysis buffer was added to the pellet, the sample was sonicated with a micro-tip sonicator^8^.

### Real-time polymerase chain reaction (RT-PCR)

A purification kit (Qiagen, 74104) was used for the isolation of RNA from mouse retinas. Five hundred nanograms of the total RNA was converted into cDNA with the SuperScript III Reverse Transcriptionase Kit (ThermoFisher Scientific, 18080093). The transcript levels were assayed in triplicate using a thermal cycler (Bio-Rad CFX96 Real Time System). The target gene was normalized to the expression level of *rpl19* using a comparative Ct method. Specific primers were as follows: *rho* (forward 5′-GCCACACTTGGAGGTGAAATC-3′, reverse 5′- AAGCGGAAGTTGCTCATCG-3′); *fas* (forward 5′-ATG AGA TCG AGC ACA ACA GC-3′, reverse 5′-TTA AAG CTT GAC ACG CAC CA-3′); *caspase 8* (forward 5′ –ATGGCGGAACTGTGTGACTCG-3′, reverse 5′-GTCACCGTGGGATAGGATACAGCA-3′); *Xbp1s* (forward 5′-GAGTCCGCAGCAGG TG-3′, reverse 5′-GTGTCAGAGTCCATGGGA-3′); *rpl19* (forward 5′-ATGCCAACTCCCGTCAGCAG-3′; reverse 5′-TCATCCTTCTCATCCAGGTCACC-3′).

### Immunohistochemistry

The superior cornea of the eyes was marked for orientation. To prepare samples for cryosectioning, the eyes were fixed in freshly prepared 4% paraformaldehyde solution overnight at 4 °C. After removal of the cornea and lens, the eyecup was washed in phosphate-buffered saline (PBS) three times, and transitioned through 5%, 10%, and 20% sucrose in PBS for 2 h each. Eyes were then embedded in an orientation-specific manner in the embedding mixture containing 1:1 ratio of Tissue-Tek embedding medium (Sakura Finetek, 4583) and 20% sucrose and then sectioned at 10-µm thickness using a cryostat. Retinal sections were blocked with 5% goat serum in PBS with 0.1% Triton-X100 (Sigma-Aldrich) and incubated with primary antibodies overnight at 4 °C. After washes, sections were incubated with secondary antibodies at room temperature for 1 h and then counterstained with ProLong Gold with DAPI (Invitrogen, P36941). Images were taken at comparable areas on sections with a fixed gain using Leica 6000 microscope (Leica Corp., Wetzlar, Germany) or a confocal microscope (Leica SP5, Leica Corp., Germany).

### Photoreceptor cell counts

The orientation marked eyes were fixed in 4% paraformaldehyde overnight followed by paraffin embedding. Six-micrometer paraffin sections were obtained using a microtome (Shandon AS325, Thermo Scientific, Cheshire, England). After deparaffinization, sections were stained with hematoxylin (Fisher Scientific, Hercules, CA) and eosin (Fisher Scientific, Hercules, CA). Only sections crossing the optic nerve were used and images were captured with Leica DM6000 microscope. Four nonoverlapping sections crossing the optic nerve from each eye were used for photoreceptor counts. The total number of photoreceptors in the whole retinal section was counted in a masked fashion.

### Quantification of GFP-LC3 puncta in photoreceptor cell inner segments

Three nonoverlapping cryosections of each GFP-LC3 mouse were used for counting of GFP-positive puncta in photoreceptor inner segments as described previously^15^. Images were taken using confocal microscope with fixed ×60 magnification. For each section, the number of GFP-positive puncta were counted in three areas of 30-µm length of retina from both superior and inferior portions. The counting area consists of the inner segment up to the first row of the photoreceptor nuclei above the outer limiting membrane. At least four animals were used for each group.

### TUNEL staining

TUNEL staining was performed on the cryosections using DeadEnd Colorimetric TUNEL System (Promega Corporation Madison, WI, USA) according to the manufacturer’s instructions. The total number of TUNEL-positive cells in the outer nuclear layer (ONL) of the whole retina section was counted and three nonoverlapping sections of each samples were used.

### Optokinetic tracking responses

The optokinetic tracking response was used to measure the visual function of the mouse as previously described. Briefly, an awake mouse was placed in a drum projected with vertical black and white stripes at various spatial frequencies. The projected drum can be rotated clockwise and counter-clockwise. Mice were monitored using a camera to track head movements (i.e, the optokinetic reflex response) in response to the rotating drum. A genotype-masked observer recorded the maximum spatial frequency (in cycles/degree (c/d)) in 100% background contrast that stimulated a tracking movement of the mouse.

### Spectral domain optical coherence tomography

Mice were anesthetized using a mixture of ketamine (80 mg/kg, Hopira, Lake Forest, IL) and xylazine (10 mg/kg, NAND, Lake Forest, IL). Pupils were dilated with topical 2.5% phenylephrine (Paragon BioTek, inc., Portland, OR) and 0.5% tropicamide (AKORN, Lake Forest, IL). After applying Systane Lubricant eye drops (Alcon. 9004494-0109) to the cornea of the mouse, optical coherence tomography (OCT) was performed using the spectral domain OCT system (Bioptigen, Inc., Durham, NC). The thickness of the ONL was measured in both superior and inferior of the retina with distances of 250 and 500 µm from the optic nerve.

### Statistical analysis

ONL thickness measured by OCT at different age points was analyzed using two-way ANOVA with replicates for multiple comparisons. For western blot, rt-PCR and other statistical comparisons across more than two experimental groups, differences were analyzed using one-way ANOVA tests followed by Tukey multiple comparison test. Comparisons between two groups were analyzed using unpaired *t*-test. Statistical analysis was performed using Prism (GraphPad, Inc., La Jolla, CA) and Microsoft Office Excel (Richmond, WA). Results were expressed as mean ± SD. Differences were considered significant at *P* < 0.05.

## Results

### Chaperone treatment improves P23H folding and decreases ERS

**Fig. 1:**
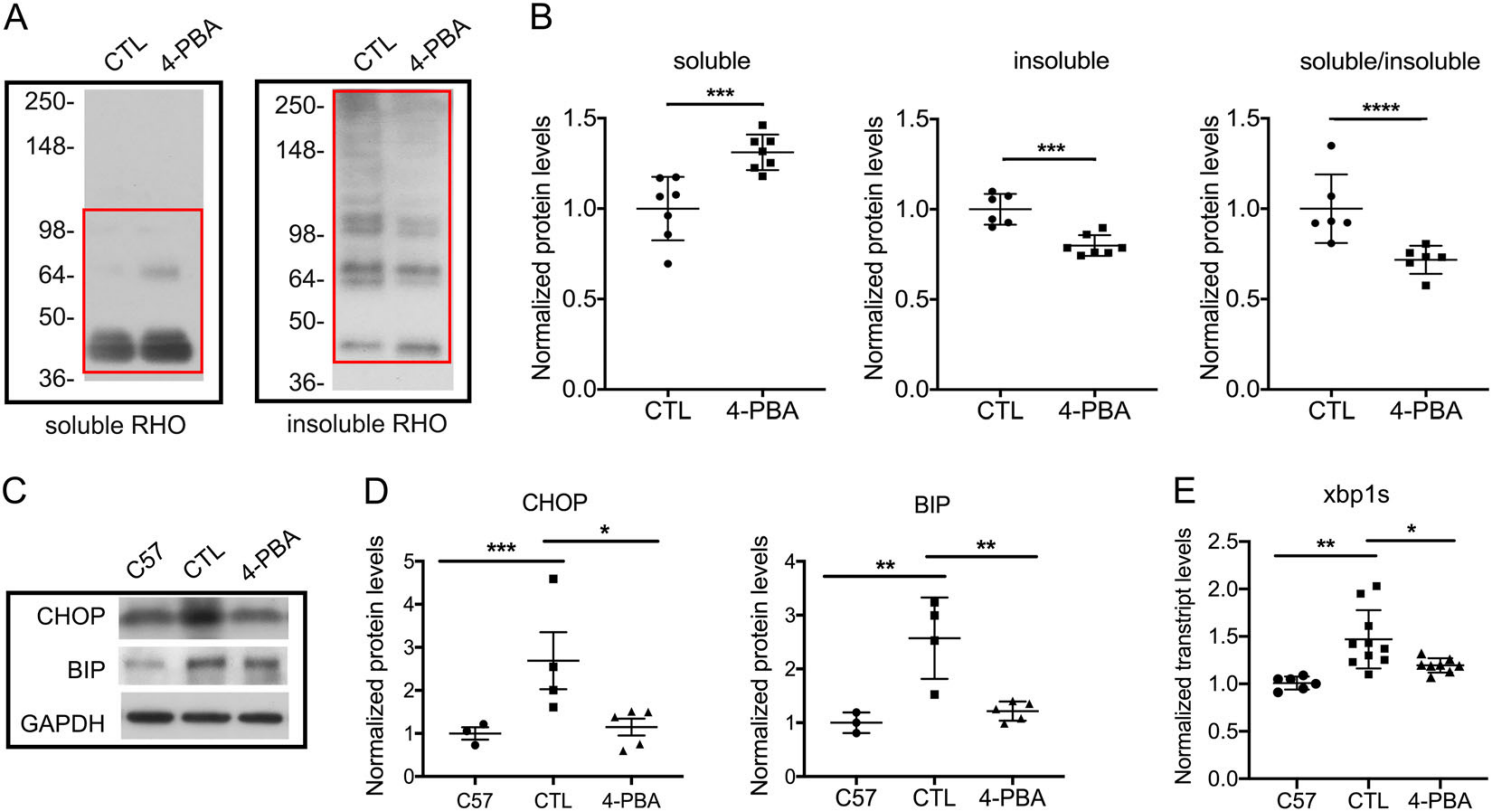
Treatment with the chaperone 4-PBA improves P23H-rhodopsin folding and reduces ERS. **a** Representative western blots probed for rhodopsin (RHO) in the soluble and insoluble fraction of total retinal protein from P23H mice after 2 weeks of treatment with 4-PBA or vehicle-only as control (CTL). **b** Quantification of western-blot band intensities of soluble and insoluble RHO normalized to total RHO, and ratio of soluble RHO to insoluble RHO. Measured areas are indicated by the red rectangles in **a**. Statistical analyses with unpaired *t*-test. **c** Representative western blots probed for CHOP, BIP, and loading control GAPDH in retinas of C57 (wild-type) and P23H mice after 2 weeks of treatment with 4-PBA or vehicle-only (CTL). **d** Quantification of the western blots represented by **c**. **e** Transcript levels of xbp1s in retinas of P23H mice treated with 4-PBA or vehicle-only (CTL) for 2 weeks, normalized to aged-matched C57 mice. Statistical analyses with one-way ANOVA. Results are shown as individual symbols representing data values along with mean and standard deviation (SD). *p ≤ 0.05. **p ≤ 0.01. ***p ≤ 0.001. ****p ≤ 0.0001

To test the hypothesis that normalizing the A:P ratio results in decreased activation of cell death pathways and increased photoreceptor function, our studies focused on the P23H mouse model of proteotoxic cell death. We began by evaluating whether improved folding and trafficking of P23H to the proteasome would result in decreased ERS and autophagy activation. P23H mice were treated with the chemical chaperone 4-PBA, which has been shown to reduce ERS both in vivo and in vitro by correcting the folding of misfolded and unfolded proteins in the ER^16–18^. The effect of 4-PBA treatment on P23H folding was evaluated by assaying its detergent-solubility, as previous studies have shown that detergent-soluble rhodopsin correlates with correctly folded protein, while detergent-insoluble rhodopsin correlates with protein aggregates^8,25^. A significant reduction in insoluble rhodopsin was observed in 4-PBA-treated P23H mice, consistent with improved rhodopsin folding and decreased aggregation (Fig. 1a, b). Analysis of the expression of proteins associated with ERS activation showed decreased levels of CHOP, BIP, and xbp1s in 4-PBA-treated mice relative to vehicle-treated controls (Fig. 1c, d, e), consistent with the conclusion that decreased ERS results from improved P23H folding.

### Improved P23H folding decreases autophagy flux

**Fig. 2:**
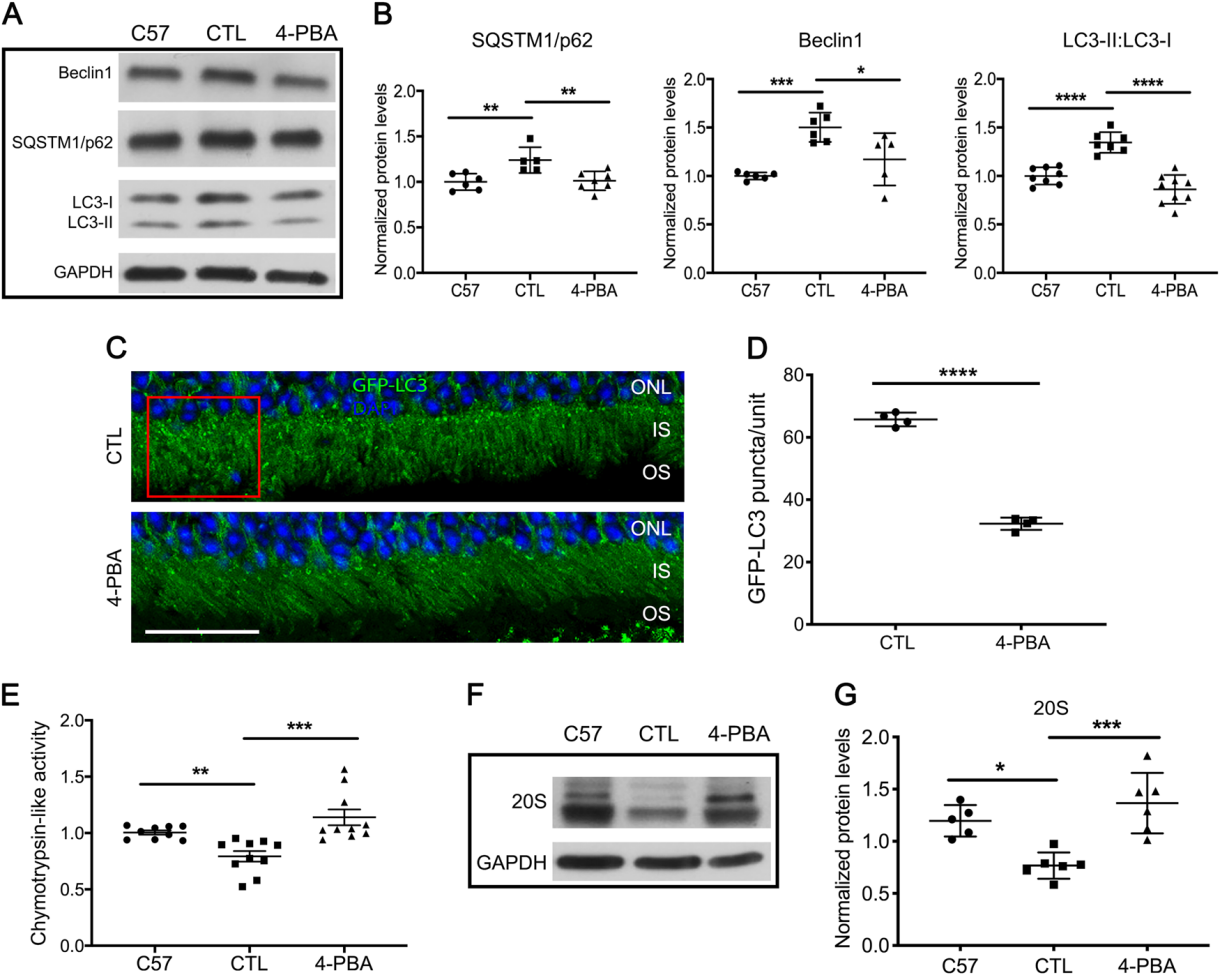
Improved P23H folding reduces autophagy flux and increases proteasome activity. **a** Representative western blots, with **b** quantification of bands probed with SQSTM1/p62, Beclin1, LC3, and loading control GAPDH in retinas of C57 (wild-type) and P23H mice after 2 weeks of treatment with 4-PBA or vehicle-only as control (CTL). Statistical analyses with one-way ANOVA. For determination of autophagy flux, animals received an intraperitoneal injection of leupeptin (40 mg/kg body weight) 4 h before tissue harvest. **c** Representative fluorescence micrographs of retinas from 1-month-old P23H GFP-LC3 mice treated with 4-PBA or vehicle-only (CTL) for 2 weeks. The GFP-LC3 puncta localize primarily to the photoreceptor inner segment (IS), with none localizing to the outer segments (OS). Nuclei of the photoreceptors in the outer nuclear layer (ONL) were stained with DAPI (blue). Scale bar: 25 µm. **d** Quantification of the number of GFP-LC3 puncta per counting unit indicated by the red square in **c**. Statistical analyses with unpaired *t*-test. **e** Chymotrypsin-like activities measured in the presence of ATP (7 µM) in retinal lysates from P23H mice treated with 4-PBA or vehicle-only (CTL) for 2 weeks, normalized to age-matched C57 (wild-type) mice. **f** Representative western blots and **g** quantification of 20S proteasome subunits and loading control GAPDH, in retinas of P23H mice treated with 4-PBA or control vehicle and untreated C57 control mice. Statistical analyses with one-way ANOVA. Results are shown as individual symbols representing data values along with mean and standard deviation (SD). *p ≤ 0.05. **p ≤ 0.01. ***p ≤ 0.001. ****p ≤ 0.0001

Activation of ERS contributes to the elimination of misfolded proteins from cells, either through the ubiquitin–proteasome or the autophagy–lysosome clearance pathways. We have previously shown that misfolded rhodopsin in the P23H retina results in chronic activation of autophagy, leading to engulfment of proteasome components by autophagosomes, and relative proteasome insufficiency^15^. To determine whether decreasing ERS in P23H mice results in decreased autophagy, the following standard measures of autophagy activation were evaluated: SQSTM1/p62, an autophagy receptor protein whose levels can serve as a metric of autophagy flux; Beclin1, an autophagy activator; and LC3-II, the lipidated form of LC3 protein^26^. As in our previous work, we observed increased SQSTM1/p62, Beclin1, and LC3-II to LC3-I ratios in the vehicle-treated P23H group compared with C57 controls (Fig. 2a, b), consistent with increased autophagy activation and autophagy flux. In contrast, retinas from P23H mice treated with 4-PBA exhibited decreased SQSTM1/p62, Beclin1, and LC3-II/LC3-I ratios, consistent with decreased autophagy flux.

Autophagy activation was also evaluated by histological assessment of LC3-positive puncta formation, as LC3-II localizes to the autophagosome membrane. To facilitate the visualization of LC3-II-coated autophagosomes, P23H GFP-LC3 mice were generated by crossing the P23H mouse with the GFP-LC3 mouse, in which LC3 is tagged with GFP^27^. Mice were then treated with 4-PBA or vehicle-only daily starting at P14. After 2 weeks of treatment, eyes were harvested for histological analysis of GFP-LC3 puncta formation. Significantly fewer GFP-LC3-positive puncta were present in the photoreceptor cells of 4-PBA-treated P23H GFP-LC3 mice, as compared with vehicle-treated P23H GFP-LC3 mice (Fig. 2c, d). These findings support the conclusion that 4-PBA treatment decreases autophagy activation and flux in the retina of the P23H mouse.

### Improved P23H folding increases proteasome activity

Proteasome insufficiency has been shown to contribute to photoreceptor cell death in the P23H retina^28^. Our previous work demonstrated that this insufficiency results, at least in part, from increased proteasome degradation due to the increased levels of autophagy induced by misfolded rhodopsin^15^. When we suppressed autophagy activation and flux, degradation of the proteasome and loss of its activity were decreased, resulting in improved photoreceptor cell survival. Given that 4-PBA decreased ERS and autophagy activation, we wanted to determine if this also rescued proteasome levels and activity. Treatment of P23H mice with 4-PBA resulted in increased chymotrypsin-like activity (Fig. 2e) and levels of 20S proteasome (Fig. 2f, g), consistent with increased proteasome activity and levels, respectively. These increases were not detected in vehicle-treated P23H mouse retinas. These findings support the conclusion that reducing autophagy activity by decreasing ERS, can relieve proteasome insufficiency and improve proteasome activity.

### Improved P23H folding decreases activation of cell death pathways

**Fig. 3:**
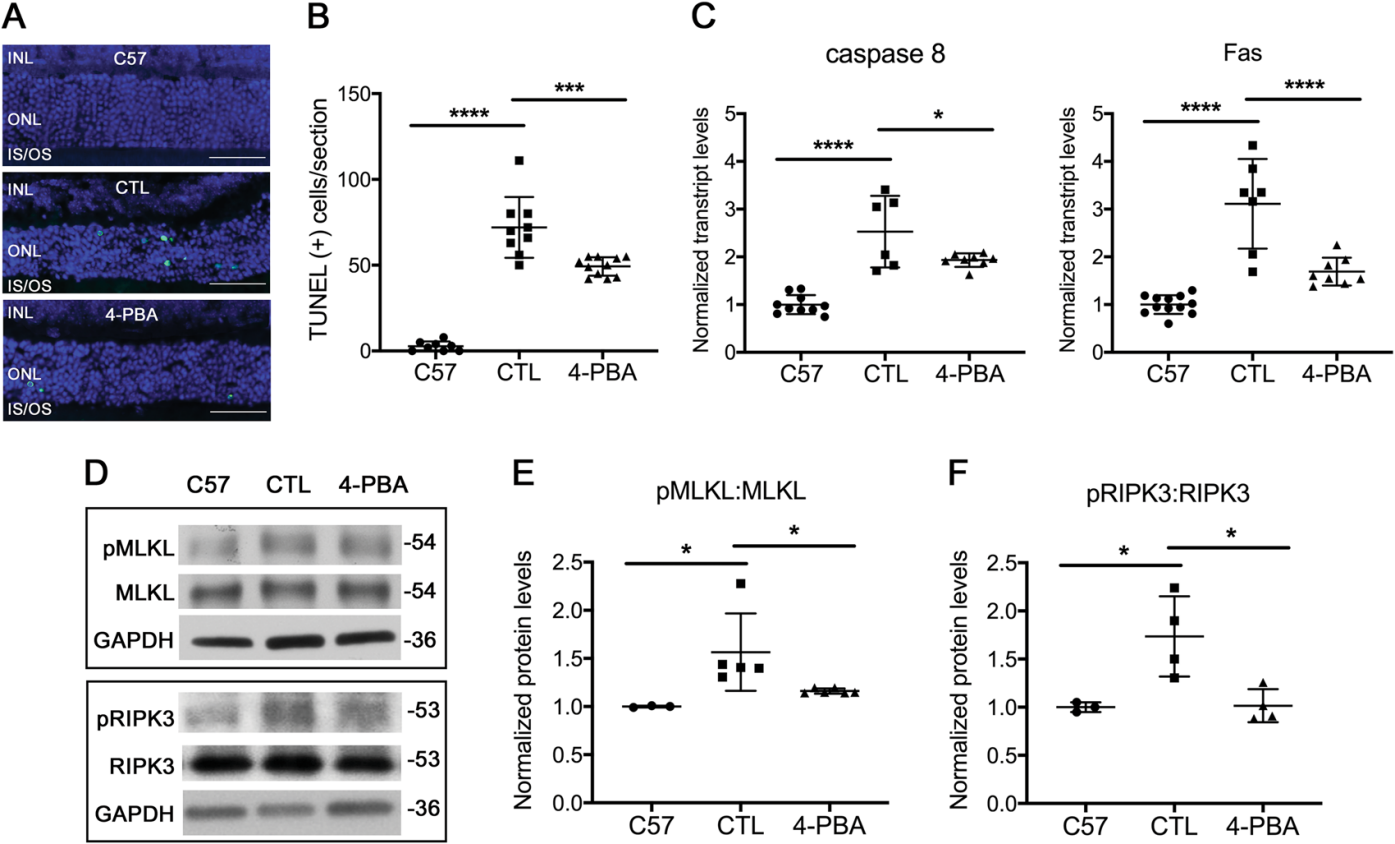
Improved P23H folding decreases activation of cell death pathways. **a** Representative TUNEL staining images and **b** quantification of number of TUNEL-positive cells from retinal sections of P23H mice treated with 4-PBA or vehicle-only as control (CTL) for 2 weeks and aged-matched C57 (wild-type) mice. Scale bar: 50 µm. Transcript levels of **c** caspase 8 and Fas-receptor in retinas of P23H mice treated with 4-PBA or vehicle-only (CTL) for 2 weeks, normalized to aged-matched C57 mice. **d** Representative western blots probed for pMLKL, MLKL, pRIPK3, RIPK3, and loading control GAPDH in retinas of C57 (wild-type) or P23H mice treated with 4-PBA or vehicle-only (CTL) for 2 weeks. Quantification of phosphorylated pMLKL (**e**) and pRIPK3 (**f**) normalized to total MLKL and RIPK3, respectively. Results are shown as individual symbols representing data values along with mean and standard deviation (SD). Statistical analyses with one-way ANOVA. *p ≤ 0.05. **p ≤ 0.01. ***p ≤ 0.001. ****p ≤ 0.0001

Both apoptosis^29,30^ and necroptosis^31^ have been reported to contribute to photoreceptor death resulting from rhodopsin misfolding in a rat P23H model. The peak of terminal deoxynucleotidyl transferase dUTP nick end labeling (TUNEL) of apoptotic photoreceptor cells in the P23H mouse retina occurs at age P15. Thus, we initiated treatment with 4-PBA or vehicle starting at P14, and assessed whether the reduction in ERS correlated with a reduction in markers of apoptotic and necroptotic cell death. Treatment with 4-PBA resulted in a decreased number of TUNEL-positive photoreceptor cells as compared with vehicle-treated controls (Fig. 3a, b). There was also a significant decrease in transcript levels of the pro-apoptotic genes Fas and caspase 8 (Fig. 3c), consistent with decreased apoptotic cell death in 4-PBA-treated mice. Decreased phosphorylation of MLKL and RIPK3, which are markers of activation of necroptosis (Fig. 3d–f), was also observed in 4-PBA-treated mice, consistent with decreased necroptotic cell death as well.

### Increased proteasome activity in the P23H retina decreases autophagy flux and cell death

**Fig. 4:**
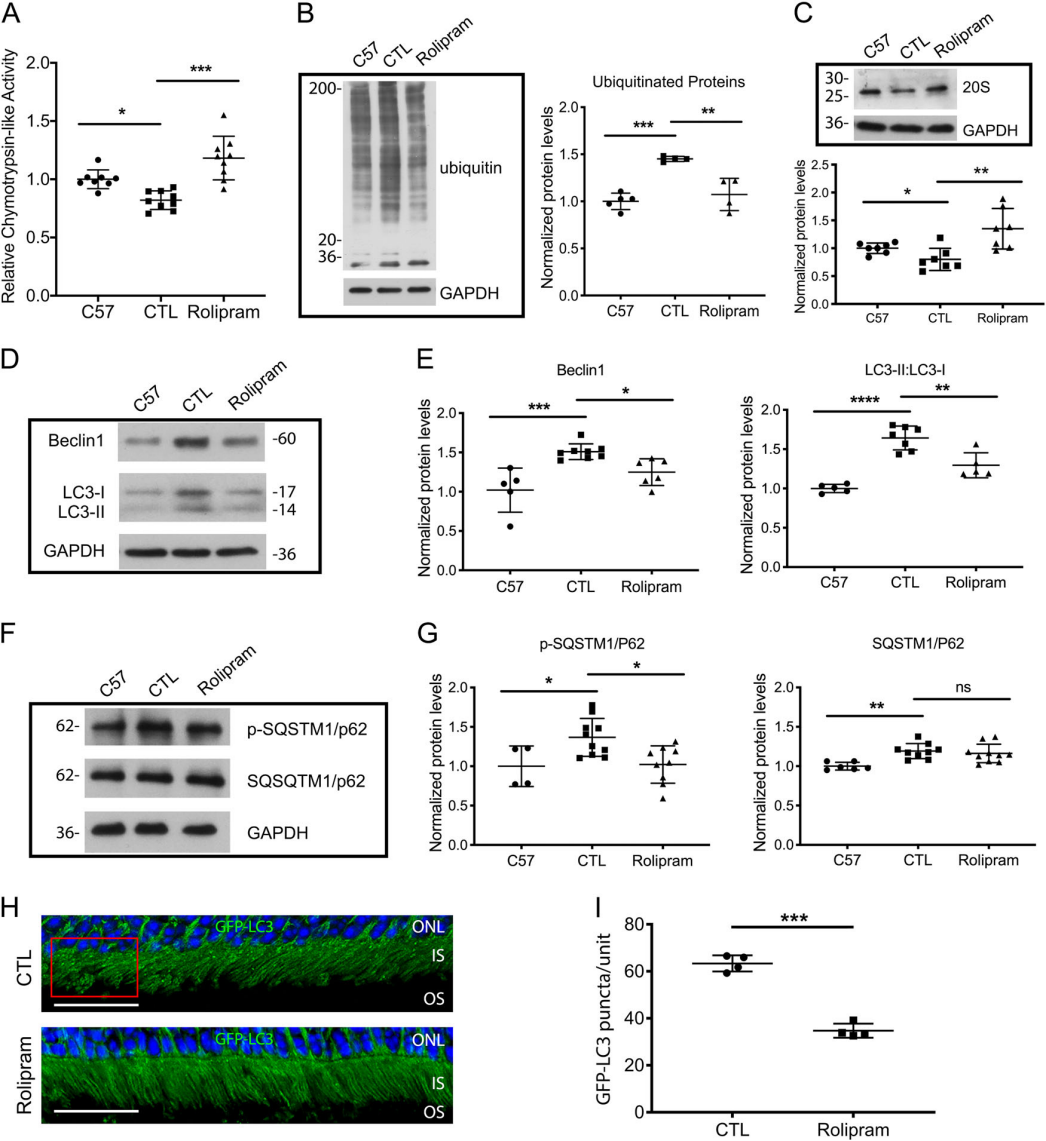
Increased proteasome activity in the P23H retina decreases autophagy flux. **a** Chymotrypsin-like activities measured in the presence of ATP (7 µM) in retinal lysates from P23H mice treated with rolipram or vehicle-only as control (CTL) for 2 weeks, normalized to age-matched C57 (wild-type) mice. Representative western blots from retinas of untreated C57 and P23H mice treated with rolipram and vehicle-only (CTL) for 2 weeks, probed and quantified for **b** ubiquitin, **c** 20S proteasome and autophagy markers **d**, **e** Beclin1, LC3, **f**, **g** phosphorylated SQSTM1/p62 and total SQSTM1/p62. To allow for determination of autophagy flux, animals received an intraperitoneal injection of leupeptin (40 mg/kg body weight) 4 h before tissue harvest. Statistical analyses with one-way ANOVA. **h** Representative fluorescence micrographs of retinas from of 1-month-old P23H GFP-LC3 mice treated with rolipram or vehicle-only (CTL) for 2 weeks. GFP-LC3 puncta localizes primarily to the photoreceptor inner segment (IS), with none localizing to the outer segments (OS). Nuclei of the photoreceptors were stained with DAPI (blue). Scale bar: 25 µm. **i** Quantification of the number of GFP-LC3 puncta per counting unit indicated in (H) as a red rectangle. Results are shown as individual symbols representing data values along with mean and standard deviation (SD). Statistical analyses with unpaired *t*-test. *p ≤ 0.05. **p ≤ 0.01. ***p ≤ 0.001. ****p ≤ 0.0001

**Fig. 5:**
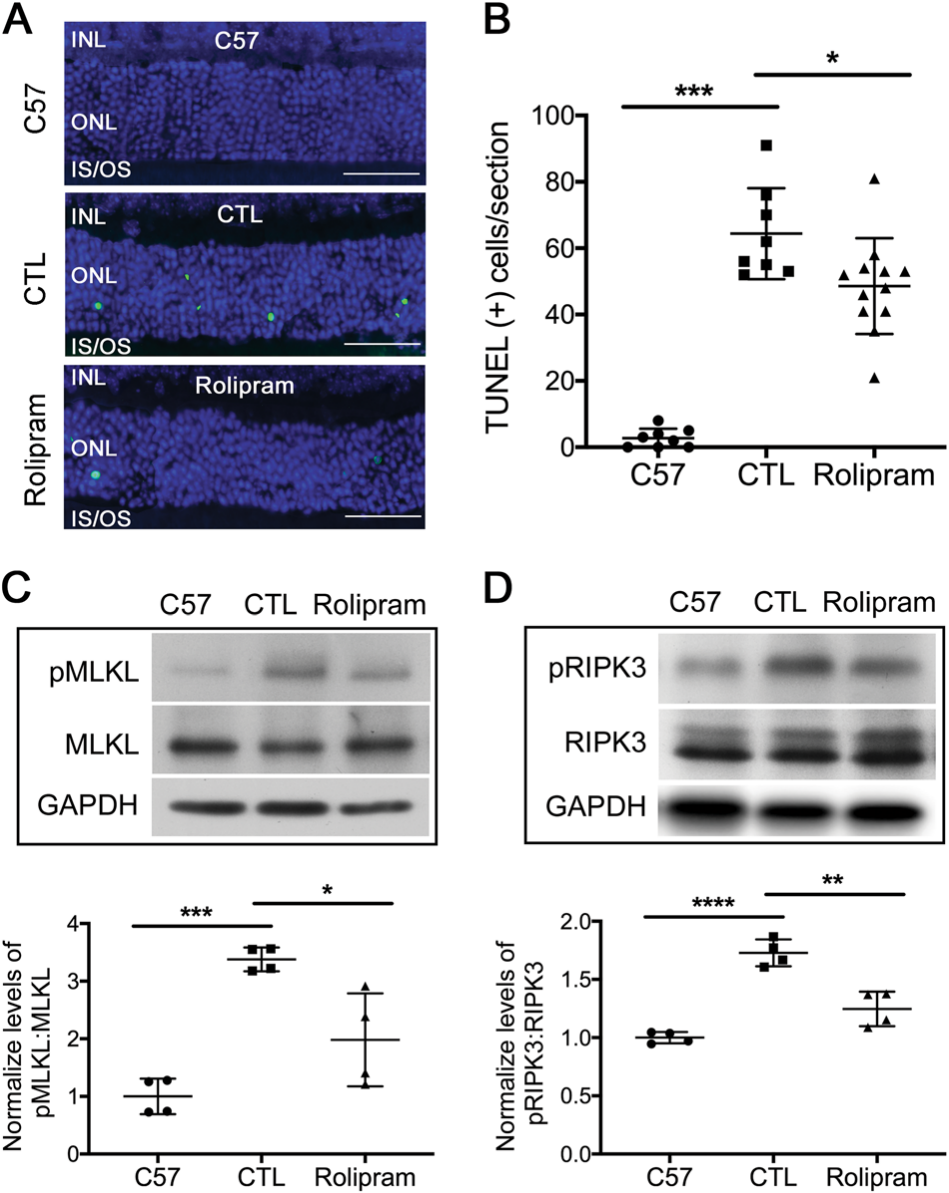
Increased proteasome activity in the P23H retina decreases photoreceptor cell death. **a** Representative TUNEL staining images and **b** quantification of TUNEL-positive photoreceptors from retinas of C57 (wild-type) mice or P23H mice treated with rolipram or vehicle-only as control (CTL) for 2 weeks. Scale bar: 50 µm. Representative western blots from retinas of C57 mice or P23H mice treated with rolipram or vehicle-only probed in **c** for pMLKL, total MLKL or GAPDH (loading control), with quantification of protein levels, or in **d** for pRIPK3, RIPK3 and GAPDH, with levels of pMLKL and pRIPK3 quantified by normalizing to levels of total MLKL and RIPK3 respectively. Results are shown as individual symbols representing data values along with mean and standard deviation (SD). Statistical analyses with one-way ANOVA. *p ≤ 0.05. **p ≤ 0.01. ***p ≤ 0.001. ****p ≤ 0.0001

Our finding that decreasing the activation of autophagy by ERS leads to improved proteasome function led us to test if the opposite was true; whether improving proteasome function results in decreased activation of autophagy. Recently, Lobanova et al. demonstrated that overexpression of proteasome subunits can decrease photoreceptor cell degeneration in the P23H retina^32^. However, in that report, no assessment of autophagy levels was made. We sought to increase proteasome function directly, through pharmacologic means, and determine the effect on autophagy activation and flux. P23H mice were treated with rolipram, a selective phosphodiesterase inhibitor that increases intracellular cAMP levels and has been shown to increase proteasome activity^19–21^. Rolipram treatment significantly increased retinal chymotrypsin-like activity in the retinas of treated P23H mice, compared with those treated with vehicle alone, with activity levels exceeding those found in the retinas of nontreated C57 mice (Fig. 4a). This increased chymotrypsin-like activity was associated with decreased levels of ubiquitinated proteins accumulating in the retina (Fig. 4b), and increased levels of the P20S subunit of the proteasome (Fig. 4c). To evaluate autophagy activation, we measured the levels of SQSTM1/p62, Beclin1, and LC3. Consistent with our hypothesis, we found significantly decreased Beclin1 and LC3-II/LC3-I in the retinas of rolipram-treated P23H mice as compared with vehicle-only-treated mice (Fig. 4d, e) Interestingly, the level of total SQSTM1/p62 did not change, but there was a decrease in the level of phosphorylated SQSTM1/p62 (p-SQSTM1/p62) (Fig. 4f, g), consistent with decreased shuttling of proteins to the autophagy pathway^33^. A corresponding decrease in the level of autophagosome formation was also seen (Fig. 4h, i). These findings support the conclusion that increasing the proteasome level and activity results in decreased autophagy activation. Consistent with this notion, we found that treatment with rolipram also decreased the number of TUNEL-positive cells in the ONL, as well as decreased the activation of necroptotic cell death (Fig. 5a–d). We interpret these findings as evidence that the normalization of the A:P ratio results in improved retinal structure and function by decreasing the activation of cell death pathways.

### Calculating the A:P ratio in treated and untreated P23H retinas

The findings described above highlight the reciprocal nature of autophagy and the proteasome, and how modulating the activity of one affects the other. In previous studies, when we measured the levels of LC3-II/LC3-I in the presence of autophagy flux blockade, we detected ~1.25-times more LC3-II accumulating in the P23H mouse retina compared with the C57 mouse retina^15^. Conversely, the P23H mouse retina showed ~0.8-times proteasome activity as compared with the C57 mouse retina (as measured by chymotrypsin-like activity). This corresponds to an A:P ratio of ~1.25/0.8 = 1.56. This calculation indicates that even small shifts in autophagy and/or proteasome activity can result in significant disruption in overall cellular homeostasis that leads to photoreceptor cell degeneration.

In this study, we replicated these results, finding an LC3-II/LC3-I ratio in the P23H retina that is ~1.34 times that found in the C57 control retina, and a proteasome activity in P23H retina that is 0.79 times that present in the C57 retina, thus resulting in an A:P ratio of ~1.69. Treatment of P23H mice with 4-PBA resulted in an A:P ratio of 0.86/1.13 = 0.76, thus dramatically decreasing the A:P ratio and bringing it closer to one. Similarly, for the retinas of P23H mice used in the rolipram treatment experiments, the vehicle-only treated group had an A:P ratio of 1.64/0.82 = 2.00, whereas the rolipram-treated animals had a much lower A:P ratio of 1.29/1.18 = 1.09. Thus, whether reducing ERS by improving P23H folding, or increasing proteasome activity directly, each modality worked to decrease autophagy activation and normalize the A:P ratio.

### Normalizing the A:P ratio in the P23H retina increases photoreceptor cell survival and function

**Fig. 6:**
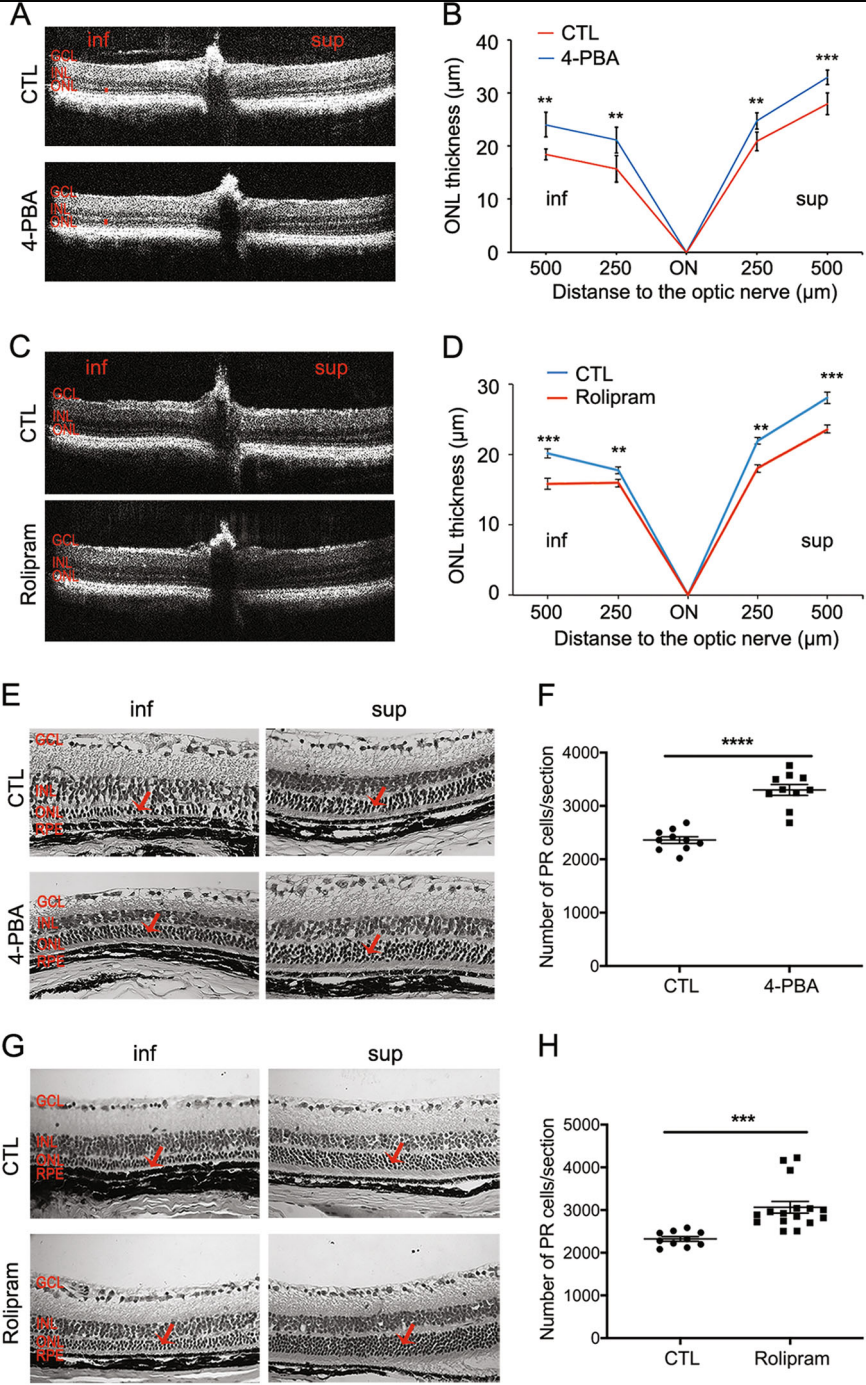
Normalizing the A:P ratio increases photoreceptor survival in P23H mice. **a** Representative optical coherence tomography (OCT) images crossing the optic nerve and **b** quantification of the outer nuclear layer (ONL) thickness of both superior and inferior retinal regions measured by (OCT) in P23H mice treated for 4 months with 4-PBA (**a**, **b**) or rolipram (**c**, **d**) or their respective vehicle-only littermates as control (CTL). Red bars in the OCT images indicate the ONL layer that was measured for each mouse. Representative H&E staining images and the number of the photoreceptors in the retinal paraffin sections from 4-PBA (**e**, **f**) or rolipram (**g**, **h**) treated P23H groups. Results are shown as individual symbols representing data values along with mean and standard deviation (SD). Statistical analyses with unpaired *t*-test. **p ≤ 0.01. ***p ≤ 0.001. ****p ≤ 0.0001

**Fig. 7:**
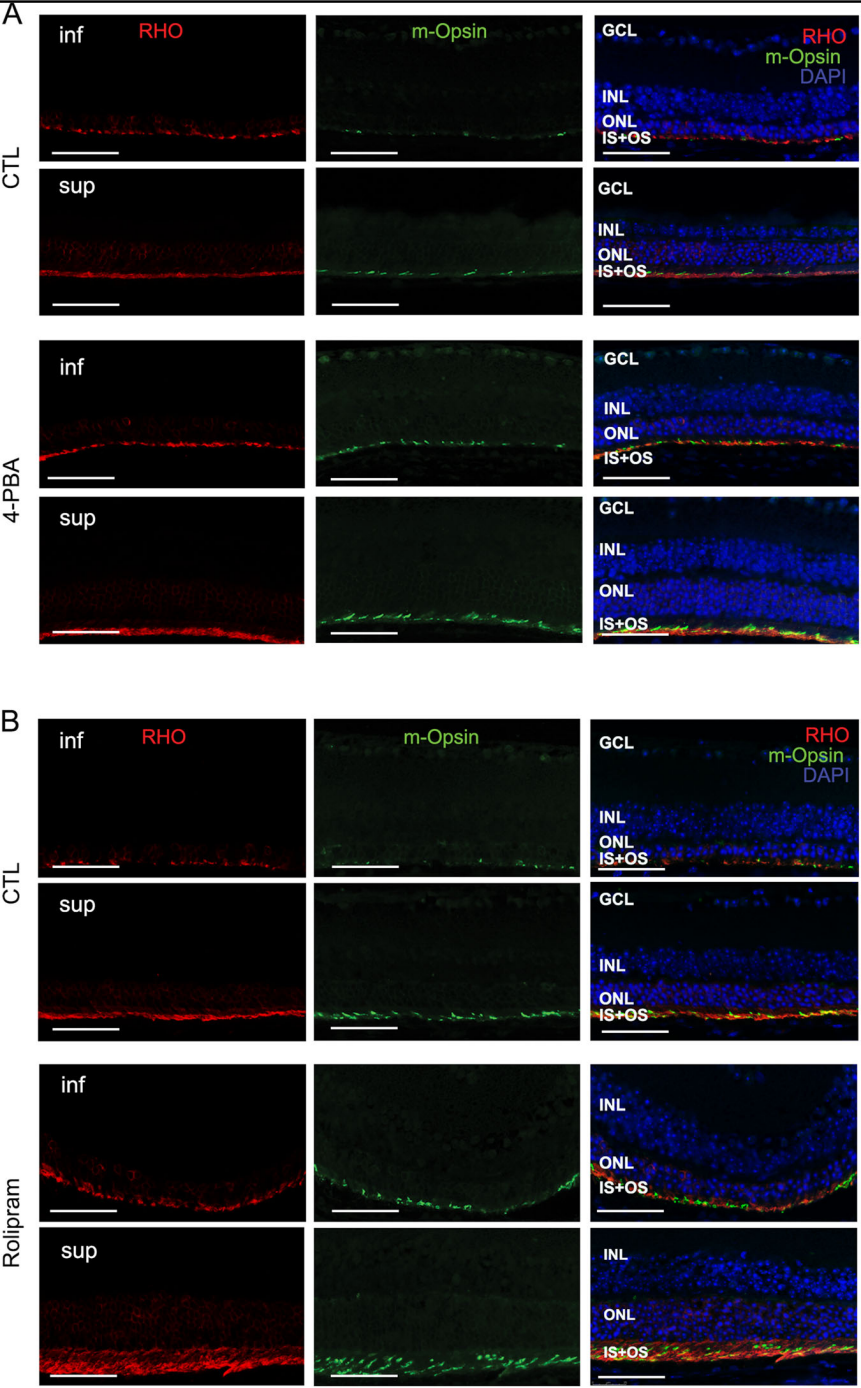
Normalizing the A:P ratio increases the expression of the opsin proteins in the photoreceptors of the P23H mice. Representative fluorescence microscopy images of retinas from of P23H mice treated with 4-PBA (**a**) or rolipram (**b**) for 4 months and their control groups, stained for rhodopsin (RHO in red), cone opsin (m-Opsin in green) and cell nuclei (DAPI in blue). Scale bar: 50 µm

**Fig. 8:**
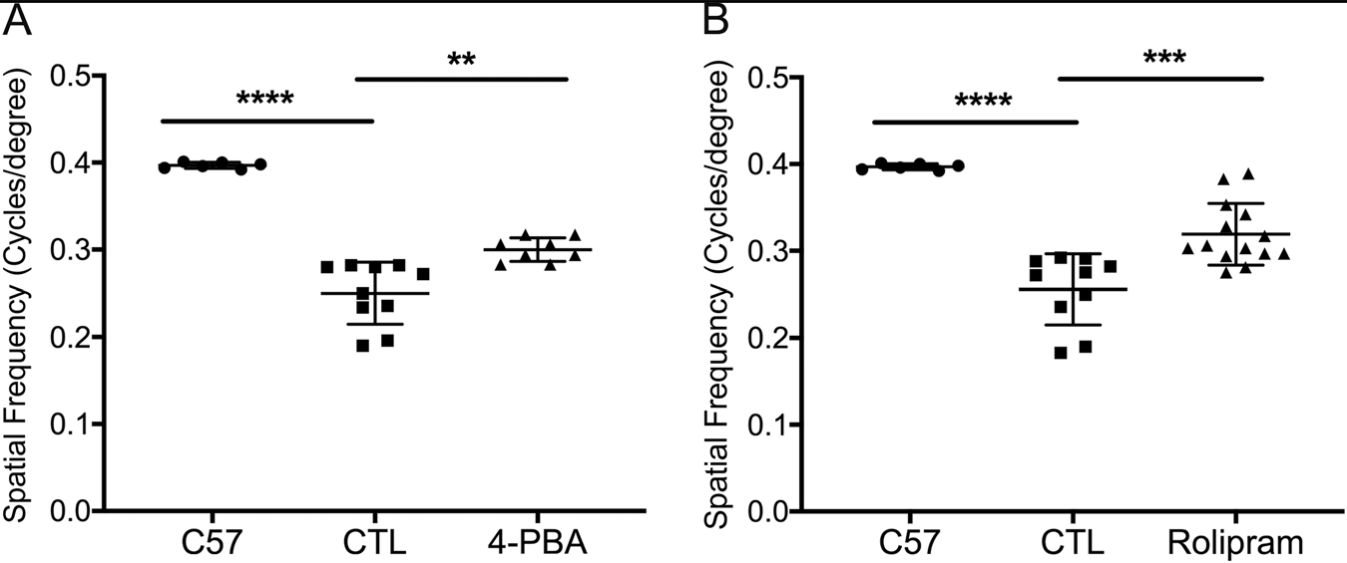
Normalizing the A:P ratio increases visual function in the P23H mice. Optokinetic tracking responses were recorded from P23H mice treated with 4-PBA (**a**) or rolipram (**b**) for 4 months, and their control groups. Responses were normalized to those of age-matched C57 (wild-type) mice. Spatial frequencies (expressed as cycles/degree) were recorded at 100% contrast. Results are shown as individual symbols representing data values along with mean and standard deviation (SD). Statistical analyses with unpaired *t*-test. **p ≤ 0.01. ***p ≤ 0.001

Ultimately, decreased activation of cell death pathways is meaningful only if it results in improved cell survival and function. In P23H mice, there is a linear rate of photoreceptor degeneration over time, with more rapid loss of photoreceptors in the inferior retina as compared with the superior retina^10^. We assessed the rate of retinal degeneration in both superior and inferior portions of the retina by measuring the ONL thickness where the photoreceptor cell nuclei reside. Treatment of P23H mice with either 4-PBA or rolipram resulted in marked preservation of ONL thickness in both superior and inferior retina as measured by OCT and compared with vehicle-treated control animals (Fig. 6a–d). This preservation of ONL was further documented by photoreceptor cell counts of HE-stained retinal sections (Fig. 6e–h). Consistent with increased photoreceptor survival, increased levels of rhodopsin and cone opsin in 4-PBA or rolipram-treated P23H mice were also detected by immunostaining (Fig. 7a, b). The visual function of 4-PBA or rolipram-treated mice versus vehicle-treated mice was analyzed using an optokinetic tracking response system. After 4 months of treatment, the optokinetic tracking responses of 4-PBA or rolipram-treated P23H mice were markedly higher than vehicle-treated controls, although lower than C57 mice (Fig. 8). These data suggest that increased photoreceptor cell preservation by 4-PBA or rolipram supports greater visual function. Taken together, our findings support the view that agents acting on different arms of the ERS–proteasome–autophagy pathways not only improve photoreceptor cell survival but also function.

## Discussion

Activation of ERS typically protects cells from protein stress^12–14^. This response is often sufficient in the context of acute perturbations, such as when transient stressors affecting translational or post-translational events result in protein misfolding or unfolding. However, in the setting of chronic protein stress, such as in the case of P23H, where a genetic mutation results in the constant production of misfolded protein, the ERS system becomes dysregulated and can no longer provide adequate protection. In previous studies we have shown that activation of ERS by misfolded rhodopsin results in dysregulated activation of autophagy^15^. This increase in autophagy activity, in turn, results in catabolism of proteasome subunits, effectively blunting the protection afforded by this complementary mechanism of protein degradation. In the present studies, we find that the ratio of autophagy to proteasome activation (the A:P ratio) in the P23H mouse retina is nearly 50% higher than the A:P ratio in the retinas of normal controls. Interventions that normalize the A:P ratio by decreasing autophagy flux relieve the proteasome insufficiency and increase photoreceptor cell survival. In addition, we demonstrate the therapeutic benefit of normalizing the A:P ratio either through interventions that decrease ERS and autophagy activation, or that directly increase proteasome capacity and activity. These outcomes establish that shifting protein degradation from autophagy to the proteasome is protective, and that acting appropriately on any of the three arms of the ERS–autophagy–proteasome relationship can serve to normalize the A:P ratio—findings that highlight the reciprocal nature of autophagy and the proteasome in regulating photoreceptor cell homeostasis.

To assess autophagy activation in the degenerating retina relative to controls, we evaluated the levels of LC3-II in the retinas of P23H and C57 mice, as LC3-II represents a physiologic marker of autophagosome abundance. It is important to note that assays of LC3-II levels during periods of high-autophagy flux result in values that are less than those obtained during periods of low- autophagy flux^26^. This is because during periods of high-autophagy flux, LC3-II is degraded by the autophagy mechanism. Thus, LC3-II levels need to be assayed under conditions that block its degradation, such as with the use of leupeptin, chloroquine or hydroxychloroquine^26^. It would also be valid to use other measures of autophagy, such as SQSTM1/p62 or the number of autophagosome puncta. The measures of autophagy activation we report are not associated with any units. They are simply a relative measure of autophagy activation in the degenerating retina compared with the wild-type control, which is a genetically similar background strain but without the IRD-causing mutation. Similarly, the assay of proteasome activity used was the chymotrypsin-like activity of the proteasome, which reflects the proteasome content of the degenerating retina relative to the control.

For visual function assessment we used the optokinetic tracking response, which serves as a quantitative measure of vision^34^. In this test, the optokinetic reflex response to vertical black-white stripes of varying spatial frequencies is measured. As the striped image is passed before the mouse, a reflex response is elicited. However, as the ability to distinguish the stripes is reduced, the reflex response becomes diminished. Thus, in degenerating retinas, the ability to elicit the reflex response to tightly spaced (high spatial frequency) stripes is reduced. Reflex responses at higher spatial frequency stripes reflect improved visual capacity. We chose this assay of vision, as it corresponds more directly to visual function than measures of electroretinogram recordings of the electrical activity of the retina. In addition, the variability of electrical activity measurements can be relatively high, even within the same animal, potentially masking differences in visual function between treatment cohorts.

Our current findings are limited to studies of retinal degeneration secondary to rhodopsin misfolding, a form of proteotoxicity in which the abnormal processing of rhodopsin results in chronic activation of the ERS and disrupted autophagy–proteasome balance. We hypothesize that the A:P ratio will also serve as a marker of altered photoreceptor cell homeostasis in retinal degenerations secondary to other forms of proteotoxicity. This may be proteotoxicity secondary to primary defects in the protein itself, or defects that result in the accumulation or aggregation of otherwise normal proteins. Examples of the former would be mutations in *RHO* that lead to rhodopsin mistrafficking, rather than misfolding. Potential examples of the latter could be mutations that disrupt transport of proteins across the photoreceptor cell connecting cilium, resulting in accumulation of normal proteins in the inner segment of the cell, leading to increased ERS.

Previous work has demonstrated the importance of apoptosis and necroptosis in photoreceptor cell death^35,36^. Effecting photoreceptor cell preservation by directly blocking these cell death pathways has remained an elusive goal, and has not yet resulted in useful neuroprotective therapies for IRD^37^. A major reason for this is that blocking one pathway of cell death often results in the shunting of the death cascade through other pathways. Our findings show that normalization of the A:P ratio reduces the activation of both the apoptotic and necroptotic cell death pathways, suggesting that altered homeostasis represented by the A:P ratio acts as an upstream activator of downstream executors of cell death. Thus, therapeutics that normalize the A:P ratio may represent a more effective strategy for the development of mutation-independent neuroprotective therapies for patients with IRD, as shunting between death pathways would be prevented. The molecular mechanism underlying the cross talk between the ERS–autophagy–proteasome axis and death-pathway activation in photoreceptor cells requires additional study, as this may represent another potential point of therapeutic intervention for decreasing retinal degeneration.

Taken together, our data demonstrate that defects in protein folding cause chronic activation of the ERS that results in imbalanced activation of autophagy relative to the proteasome, which in turn promotes photoreceptor cell death. It follows that measures of the relative activity of autophagy versus the proteasome, which we have designated as the A:P ratio, represent an important readout, or biomarker, will be important in informing the development of mutation-independent therapies aimed at reducing proteotoxicity in IRD. We propose that the A:P ratio represents a measure of homeostasis within photoreceptor cells, and that treatments designed to normalize this important gatekeeper of cellular homeostasis have the potential to decrease retinal cell death in IRD. We predict that this concept can be extended beyond photoreceptor cells, and represents a novel perspective for assessing proteotoxicity across a spectrum of disease states.
